# The potential impact of the next influenza pandemic on a national primary care medical workforce

**DOI:** 10.1186/1478-4491-3-7

**Published:** 2005-08-11

**Authors:** Nick Wilson, Michael Baker, Peter Crampton, Osman Mansoor

**Affiliations:** 1 Department of Public Health, Wellington School of Medicine & Health Sciences, Otago University, Wellington, New Zealand; 2 Public Health Consulting Ltd, Wellington, New Zealand

## Abstract

**Background:**

Another influenza pandemic is all but inevitable. We estimated its potential impact on the primary care medical workforce in New Zealand, so that planning could mitigate the disruption from the pandemic and similar challenges.

**Methods:**

The model in the "FluAid" software (Centers for Disease Control and Prevention, CDC, Atlanta) was applied to the New Zealand primary care medical workforce (i.e., general practitioners).

**Results:**

At its peak (week 4) the pandemic would lead to 1.2% to 2.7% loss of medical work time, using conservative baseline assumptions. Most workdays (88%) would be lost due to illness, followed by hospitalisation (8%), and then premature death (4%).

Inputs for a "more severe" scenario included greater health effects and time spent caring for sick relatives. For this scenario, 9% of medical workdays would be lost in the peak week, and 3% over a more compressed six-week period of the first pandemic wave. As with the base case, most (64%) of lost workdays would be due to illness, followed by caring for others (31%), hospitalisation (4%), and then premature death (1%).

**Conclusion:**

Preparedness planning for future influenza pandemics must consider the impact on this medical workforce and incorporate strategies to minimise this impact, including infection control measures, well-designed protocols, and improved health sector surge capacity.

## Background

It is probably only a matter of time before the next influenza pandemic. The only uncertainties are its timing and impact. Effective planning for public health interventions before and during a pandemic is likely to reduce its impact [1]. A pandemic is likely to be extremely disruptive, particularly for the health sector. Not only will there be a surge in demand for health services (preventive as well as curative), but the health workforce is likely to have higher exposure and incidence rates.

We estimated for the impact of pandemic influenza on the primary care medical workforce (i.e., general practitioners) for a single country – New Zealand. The estimates inform planning for the pandemic as well as for other new emerging infectious disease threats, including those from bioterrorism, by providing some estimates for the level of "surge capacity" that must be built into the health sector.

New Zealand has approximately 23 000 health practitioners plus around 30 000 support workers delivering services in the community [2]. Around 40% of its medical practitioners and 23% of its nurses work in primary care settings. The population to general practitioner (GP) ratio varies considerably across different territorial authorities from about 450 to about 2300 [3]. New Zealand's Primary Health Care Strategy [4], released in 2001, places primary care at the centre of the country's health system. It defines a future for primary care where, increasingly, primary care and public health strategies are expected to be coordinated and intermeshed, with the overall objective of improving population health and reducing health inequalities.

The Strategy led to the formation of new non-profit umbrella organisations, called Primary Health Organisations (PHOs) [5]. PHOs are responsible for ensuring that their constituent general practices and community organisations provide comprehensive, continuing and coordinated care to their enrolled populations, including health promotion and prevention programmes. Increasingly, PHOs are held accountable to their funders for a range of population health outcomes. The development of PHOs mirrors, to an extent, the development over the past five years of primary care groups and trusts in the United Kingdom [6].

## Methods

### The FluAid model

The United States Centers for Disease Control and Prevention (CDC) have developed a relatively simple deterministic model, "FluAid" (on freely available software), for analysing the impact of future influenza pandemics [7]. The output of the model is the number of deaths, hospitalisations, and illnesses requiring medical consultations for a wave of pandemic influenza. These outputs were used to estimate lost workdays in this analysis. The model assumes that no public health interventions (e.g., limitations of movement, vaccine, or antiviral drugs) are used to control disease spread. Specific details on the FluAid software and the various assumptions in the model are detailed on the CDC website [8] and other documents [9,10]. This model has been used in other studies [11-13].

### Baseline model assumptions

The default values used in FluAid were used for the mortality rates, the hospitalisation rates and the rates of illness. The default values for the incidence rates of clinical illness were also used (i.e., 15% and 35% for "most likely" results). Working doctors are likely to be in better health than the general population (the "healthy worker effect"), and have fewer risk factors associated with severe sequelae from influenza infection. For example, they are probably far less likely to have chronic respiratory disease, since they have markedly lower smoking rates in New Zealand than the rest of the population [14]. Therefore, the proportion of doctors assumed to be in the "high-risk" category was arbitrarily halved (i.e., from 14.4% for 19–64-year-olds down to 7.2%). This may be overly conservative, but we wished to systematically err towards underestimating the impact of a pandemic on this workforce in the baseline model.

The length of time associated with hospitalisation (average of eight days) and from clinical illness (two days) was based on the United States data in a previously published model [10]. In addition to this, it was assumed that there would be one day of convalescence for clinical illness and three days convalescence after hospitalisation (i.e., before returning to work). To determine the *working days *lost, these figures were adjusted by the proportion of a typical week that is spent at work (i.e., five out of seven days).

### Population data

The latest available national figure for the total number of registered medical practitioners working in primary care was 3074 (i.e., those working four or more hours per week in 2002 and who are classified as working in "general practice" or "primary care") [15]. The average hours worked per week by these doctors is 42 hours, and it was assumed that they would work full time during the pandemic period (unless affected by illness).

### Time distribution

The FluAid model does not consider the time frame of the pandemic within an affected region. The length of influenza epidemics is highly variable [16,17]. For the baseline analysis the distribution of cases and a duration of eight weeks was used, based on the results of a stochastically simulated influenza pandemic [18].

### "More severe" scenario assumptions

For this scenario the pandemic wave was assumed to last only six weeks and the upper range "maximum" values from the FluAid model were used (for the 35% incidence rate scenario). The proportion of cases in the peak week was raised to 40% (from 32.3%), the upper limit of the days of hospitalisation was used (13 days [10]), and days lost from illness was doubled relative to the baseline model (i.e., to four days). In addition, it was assumed that every doctor would spend an average of 0.5 days during the pandemic wave period caring for sick relatives or household members.

## Results

Baseline assumptions result in 584 to 1320 lost workdays for 15% and 35% incidence rates respectively (Table 1). The lost work time was 1.2% to 2.7% of maximal capacity at the time of estimated peak pandemic impact (week 4). An estimated 88% of lost workdays arose from illness not requiring hospitalisation, 8% from hospitalised cases, and 4% from deaths caused by influenza (when using the 35% incidence rate).

**Table 1:** Predicted impact of pandemic influenza on the population of active and registered primary care medical practitioners based on modelling with FluAid (n = 3074 doctors, 15% and 35% incidence rates)

**Week of pandemic in NZ**	**Deaths (No.)**	**Hospital – isations (No.)**	**Illnesses (No.)^a^**	**Lost workdays from deaths (No.)^b^**	**Lost workdays from hospitalisations (No.)**	**Lost workdays from illness (No.)^a^**	**Total lost workdays (No.)**	**Reduction in days worked (%)^c^**
1	0.0	0	5 – 11	0	1 – 2	10 – 23	11 – 25	01 – 0.2%
2	0.1	1	26 – 61	1	5 – 11	56 – 130	62 – 142	0.4 – 0.9%
3	0.2	1 – 3	59 – 137	2	12 – 25	126 – 294	139 – 321	0.9 – 2.1%
4	0.3	2 – 4	77 – 180	3	15 – 33	165 – 386	184 – 422	1.2 – 2.7%
5	0.2	1 – 2	43 – 101	4	9 – 19	93 – 217	106 – 240	0.7 – 1.6%
6	0.1	1 – 1	20 – 47	5	4 – 9	44 – 101	52 – 115	0.3 – 0.7%
7	0.0	0	5 – 12	5	1 – 2	11 – 26	17 – 33	0.1 – 0.2%
8	0.0	0	3 – 7	5	1	7 – 16	12 – 22	0.1%

Total^d^	1	6 – 13	239 – 556	25	47 – 102	512 – 1192	584 – 1320	0.5 – 1.1%

^a^For those with clinical illness that is severe enough to require a medical consultation – but which does not result in hospitalisation.

^b^The impact is cumulative for deaths in terms of lost workdays.

^c^Relative to the full workforce working for five days per week.

^d^The figures in the columns do not add up exactly to the totals due to rounding.

The "more severe" model inputs resulted in 3591 lost workdays (Table 2), with a loss of 9.3% of maximal capacity in the peak week and 2.9% over the six-week period. The lost workdays mainly arose from the impact of illness not requiring hospitalisation (64%), then the time spent caring for others (31%), the impact of hospitalisation (4%), and then the impact of premature death (1%).

**Table 2:** Predicted impact of pandemic influenza on the population of active and registered primary care medical practitioners using "more severe" scenario assumptions and based on modelling with FluAid (n = 3074 doctors)

**Week of pandemic in NZ**	**Deaths (No.)**	**Hospital-isations (No.)**	**Illness (No.)^a^**	**Lost workdays from deaths (No.)^a^**	**Lost workdays from hospitalisations (No.)**	**Lost workdays from illness (No.)^a^**	**Lost workdays from caring for others**	**Total lost workdays (No.)**	**Reduction in days worked (%)^a^**
1	0.2	1	64	1	13	184	88	285	1.9%
2	0.4	3	161	3	32	459	220	713	4.6%
3	0.8	6	321	7	64	918	439	1428	9.3%
4	0.4	3	161	9	32	459	220	719	4.7%
5	0.2	1	64	10	13	184	88	294	1.9%
6	0.1	1	32	10	6	92	44	152	1.0%

Total*	2	14	803	39	160	2294	1098	3591	2.9%

^a^See footnotes for Table 1.

## Discussion

### Impact on health and workdays

The model results suggest a substantial impact on general practitioners, even with very conservative assumptions. For the "more severe" scenario a mortality rate of 65 per 100 000 is predicted (albeit for just one pandemic wave). This is much less than the total population rate for the 1918 pandemic in New Zealand of 745 per 100 000 [19], but it is more than United States total population rates for the 1957 Asian flu pandemic (22 per 100 000) and the 1968 Hong Kong flu pandemic (14 per 100 000) [20].

The results suggest that the major contributor to lost workdays will be episodes of uncomplicated illness that do not require hospitalisation. If time spent caring for sick relatives is considered (i.e., as in the "more severe" scenario) then this also made a substantial contribution to the total workdays lost. The impact of lost workdays will be magnified by the increased demand on the medical workforce, as has recently been modelled for primary care consultations and hospitalisations in New Zealand [12], and for critical care services in both New Zealand and Australia [21].

### Implications for the health sector

There are several broad strategies to reduce the impact of an influenza pandemic on health care workers. First, infection control strategies aimed at doctors need to be in place. These measures include basic hygiene practices and also mask use may be appropriate (depending on risk [1]). Health authorities and doctors themselves could also stockpile and then use antivirals at the appropriate time. Such stockpiling has already commenced at a national level in New Zealand and various other countries [22]. Recent modelling work indicates that access to enough antivirals could substantially reduce the number of clinical cases and hospitalisations in the population [23].

Second, pandemic planning needs to include specific measures to maintain the functional capacity of health care workers, bearing in mind that the impact of an influenza pandemic is likely to vary between urban and rural areas. While exposure to infection may be less in relatively isolated rural areas, such areas generally have far less "spare" health care capacity, should GPs be incapacitated. General practices and health authorities can consider plans to provide care for the ill dependents of their medical staff so as to reduce absenteeism rates. Through other pandemic planning activities they can also potentially reduce the overall impact of a pandemic and hence demands on their staff. For example, rapid action at the start of the pandemic to cancel elective procedures could enhance workforce capacity. Establishing dedicated primary care assessment centres for patients with suspected influenza could also reduce overall GP workload.

Third, strategies are needed to manage the psychological impact of pandemic influenza on health care workers. Surveys of such workers show that they report a lower willingness to report for duty for infectious diseases epidemics (SARS, smallpox) than for most other forms of catastrophic disasters (environmental disasters, mass casualty incidents) [24]. Experience with SARS also demonstrated the psychological importance of having well-designed policies and protocols in place. Even in situations where health care workers perceive themselves to be at increased risk, they report feeling reassured by simple protective measures based on sound epidemiological principles, when implemented in a timely manner [25]. A review of the foundations for a SARS preparedness and response plan has specifically highlighted the importance of both appropriate staffing and support [26].

Fourth, improving health sector surge capacity now would be desirable as the New Zealand health sector is often running at stretched capacity (e.g., especially emergency departments [27]). Expanding existing services such as the "Healthline" (a free telephone information service to the public staffed by nurses) may also be worthwhile. Similarly, active promotion of key websites with information on managing influenza (e.g., as per the CDC website [28]) could be publicised each winter season. All such measures would benefit the public prior to a pandemic as well as potentially reducing the demands on the medical workforce in the primary care and secondary care settings during a pandemic.

Finally, a greater focus on the primary care nursing workforce would be of benefit. Following the implementation of the Primary Health Care Strategy there has been a rapid shift to capitation funding of general practices, and an attendant increased focus on team-based primary care (principally GPs and practice nurses). This trend raises the possibility of increasing substitution of GP work roles by nurses. This type of substitution has occurred for a decade or more in a range of community-governed non-profit practices and other capitation-funded practices [29,30]. A recent review of the medical workforce in New Zealand also highlights the potential efficiencies from some role shifting from doctors to other health workers [31]. Expanding such a non-medical health workforce, while also vulnerable to the infection during a pandemic, would provide a buffer for the GP workforce in the event of attrition of GP capacity.

### Limitations with the modelling

The uncertainties associated with pandemic influenza mean that estimating its future impact is problematic. This model could substantially underestimate the true impact because the new strain may be particularly infectious and/or virulent, and the incidence rate for clinical illness might be higher for doctors given their likely occupational risk [32]. For example, one review of nosocomial outbreaks reported a health care worker incidence rate to be as high as 60% [33]. Furthermore, doctors may be relatively slow to seek care for themselves – especially at the time of a national crisis when their professional obligations are greatest. Other parameters used in the modelling may also have been overly conservative, such as the extent of the healthy worker effect among doctors and the amount of time off work taken to care for sick relatives (which was zero in the baseline model and fairly small at 0.5 days in the "more severe" scenario). There was also no consideration in the model of absenteeism effects from fear of infection (e.g., in the case of particularly virulent strains). Indeed, this absenteeism effect could be more important than actual disease in reducing health sector capacity.

Although we consider that the baseline results are more likely to underestimate than to overestimate the impact of a future influenza pandemic, there are still plausible reasons why they could be overestimates. These include the following:

• various international and national public health interventions (as recommended by WHO [1]) may reduce the impact of pandemic influenza;

• at least for subsequent pandemic waves, an appropriate vaccine may be available;

• antivirals could prevent infection and reduce morbidity amongst the medical workforce and the rest of the population [23];

• improved treatment could lower hospitalisation and mortality rates (relative to the figures used in this model).

### Further research

This modelling could be further refined to address some of the limitations detailed above. Clarifying the prevalence of "high-risk" conditions among the medical workforce would be a particularly important refinement along with improving the estimates of time off work to care for relatives (or even absenteeism from fear of infection). Expanding such modelling to other parts of the health sector workforce is also desirable, along with exploring the extent that such research is generalisable to other threats (e.g., from other new emerging infectious threats, including those from bioterrorism).

## Summary

This modelling work has a number of limitations and so these results could still substantially overestimate or underestimate the impact of the next influenza pandemic on the primary care medical workforce. Nevertheless, this modelling work highlights the importance of infection control strategies for health care workers, pandemic planning, and improving current health sector surge capacity.

## Competing interests

The author(s) declare that they have no competing interests.

## Authors' contributions

All authors contributed to the writing and NW, MB and OM also contributed to the design. NW conducted the analyses. All authors read and approved the final manuscript.
